# Molecular Cloning, Characterization and mRNA Expression of a Chitin Synthase 2 Gene from the Oriental Fruit Fly, *Bactrocera dorsalis* (Diptera: Tephritidae)

**DOI:** 10.3390/ijms140817055

**Published:** 2013-08-19

**Authors:** Li Chen, Wen-Jia Yang, Lin Cong, Kang-Kang Xu, Jin-Jun Wang

**Affiliations:** Key Laboratory of Entomology and Pest Control Engineering, College of Plant Protection, Southwest University, Chongqing 400716, China; E-Mails: chenli420625@gmail.com (L.C.); yangwenjiaxkk@gmail.com (W.-J.Y.); iamconglin820@gmail.com (L.C.); xukangkangywj@gmail.com (K.-K.X.)

**Keywords:** *Bactrocera dorsalis*, chitin synthase 2, cDNA cloning, expression profiles, midgut, chitin content

## Abstract

Chitin synthase (CHS), a potential target for eco-friendly insecticides, plays an essential role in chitin formation in insects. In this study, a full-length cDNA encoding chitin synthase 2 (*BdCHS2*) was cloned and characterized in the oriental fruit fly, *Bactrocera dorsalis*. The *BdCHS2* cDNA had 4417 nucleotides, containing an open reading frame of 4122 nucleotides, which encoded 1373 amino acid residues with a predicted molecular weight of 158.5 kDa. Phylogenetic analysis with other insect CHSs suggested that *BdCHS2* belongs to insect CHS2. The *BdCHS2* transcript was predominately found in midgut but was detected at low levels in fat body, Malpighian tubules, integument, and trachea. Moreover, *BdCHS2* was expressed in all developmental stages, and highly expressed in the feeding stages. There was a positive relationship between *BdCHS2* expression and total chitin content during development. Furthermore, both the gene expression and chitin content in midgut decreased when the insect was fed for 24 h, then starved for 24 h, while they increased dramatically and rapidly under the condition of starvation for 24 h then feeding for 24 h. These results suggest that *BdCHS2* may play an important role in regulating chitin content of the midgut, and subsequently affect the growth and development of *B. dorsalis*.

## 1. Introduction

The oriental fruit fly, *Bactrocera dorsalis* (Hendel), is one of the most damaging horticultural pests in Asian and Pacific countries [1], causing enormous losses in a wide variety of fruits and vegetables [2]. In recent years, it has become an especially troublesome pest because of its ability to develop resistance to various insecticides [3,4]. Therefore, more potential and powerful approaches are urgently needed for *B. dorsalis* control.

Chitin, widely distributed in fungi, nematodes and arthropods, is an especially abundant natural biopolymer, second only to cellulose. It is an important structural component of the insect trachea, cuticle, cuticular lining of the foregut, hindgut, and peritrophic membrane (PM) that lines the lumen of the midgut [5,6]. Chitin is a linear polymer of β-(1,4)-*N*-acetyl-d-glucosamine (GlcNAc), which plays a key role in protecting insects against external invasion of microorganisms, and the abrasion of food [7]. Based on the site of synthesis, the PM has two types: type I PM is only formed in response to feeding and the type of meal ingested which delaminated from the entire midgut epithelium (e.g., Coleoptera, Orthoptera, and larval Lepidoptera); type II PM presented throughout the life cycle is produced by a specialized tissue at the anterior midgut (e.g., Dermaptera, Isoptera, and larvae of Diptera) [6]. The presence of the chitin in the insect cuticle and the PM as well as the absence of chitin in plants and animals make chitin a potential selective target for insect control.

Chitin synthase (CHS) is a critical enzyme for synthesis of chitin and thus for subsequent growth and development in insects. It belongs to a large family of glycosyltransferases that catalyze the transfer of sugar moieties from activated sugar donors to specific acceptors resulting in a glycosidic bond [5,7]. Insect chitin synthases can be classified into two different types: CHS1 and CHS2. These two chitin synthases are very close to each other and have some basic properties in common. In the catalytic center, the two chitin synthases share some conserved motifs such as “DXD”, “EDR”, “CATMWHXT” and “QRRRW” which contribute to divalent cation binding, catalysis, and substrate binding, respectively [7]. During insect growth and development, *CHS1* and *CHS2* have different functions. *CHS1* is predominantly expressed in the epidermis and tracheal cells that are responsible for chitin synthesis in cuticle and trachea [8]. *CHS2* is mainly expressed in the midgut and is presumably responsible for synthesizing the chitin in the PM at the feeding stage [9,10]. However, a recent study showed that both enzymes were detected in newly formed compound eyes of *A. gambiae* pupae by using immunohistochemical analysis [11]. Moreover, *CHS2* has no alternative splicing variants, whereas *CHS1* is known to have alternative exons, producing two splicing variants. To date, the genes encoding CHS2 protein have been characterized in several insect species, including *Aedes aegypti* [12], *Drosophila melanogaster* [13], *Tribolium castaneum* [14], *Manduca sexta* [15], *Spodoptera exigua* [10], *Ostrinia furnacalis* [16], *Spodoptera frugiperda* [9], *Locusta migratoria* [17], and *Anopheles gambiae* [11]. The insect CHSs have received much attention and represent potential targets for developing selective insecticides.

A few studies showed that feed-mediated conditions played a role for gut CHS in controlling chitin-content, including the expression level of the *CHS2* gene; chitin contents were changed by insect feeding or not [18,19]. If this gene is involved in the nutrient processing in midgut, the PM will be a candidate target site in pest management for disrupting the function to decrease the efficiency of the digestive process [18]. The part of chitin in the old cuticle needs to be digested followed by the synthesis of chitin for the formation of new cuticle during molting. Inhibition of CHS2 activity will result in insect death due to starvation [20].

In this study, we reported cloning and characterization of a chitin synthase 2 gene (*BdCHS2*) from *B. dorsalis*. The expression patterns of *BdCHS2* at various developmental stages and in different tissues of the third instar larvae were examined. Moreover, feeding-mediated changes in transcription levels of *BdCHS2* were also investigated, and correlations of *BdCHS2* expression and chitin content in the midgut of *B. dorsalis* were analyzed.

## 2. Results and Discussion

### 2.1. Identification and Characterization of *BdCHS2*

**Figure 1:**
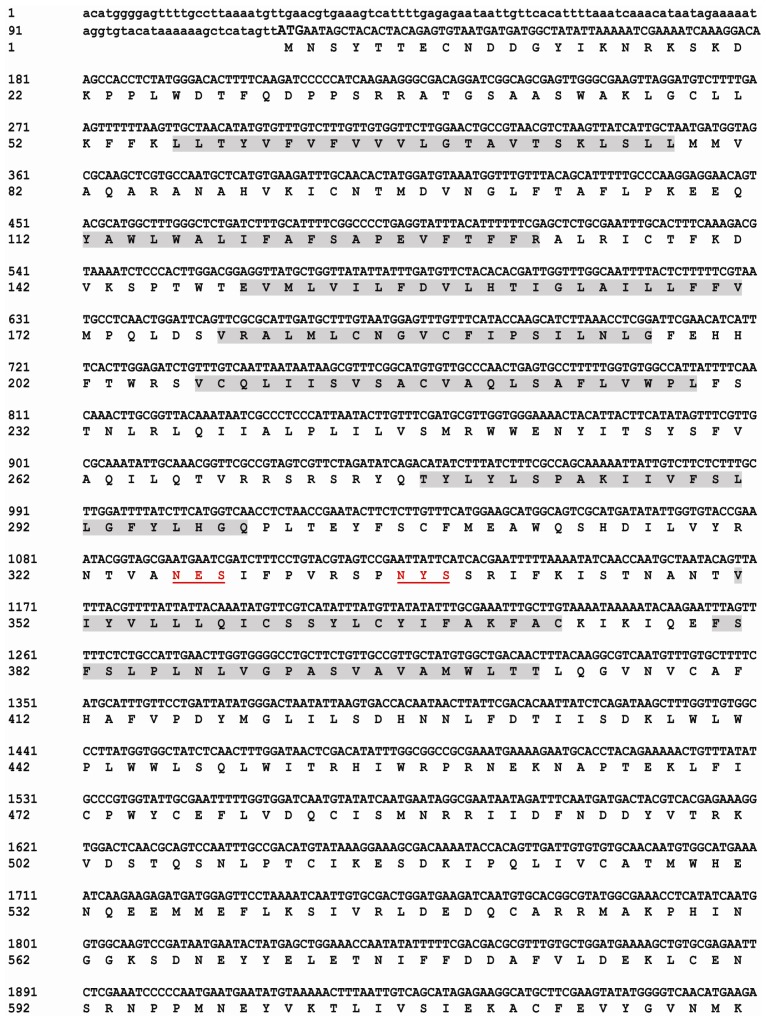

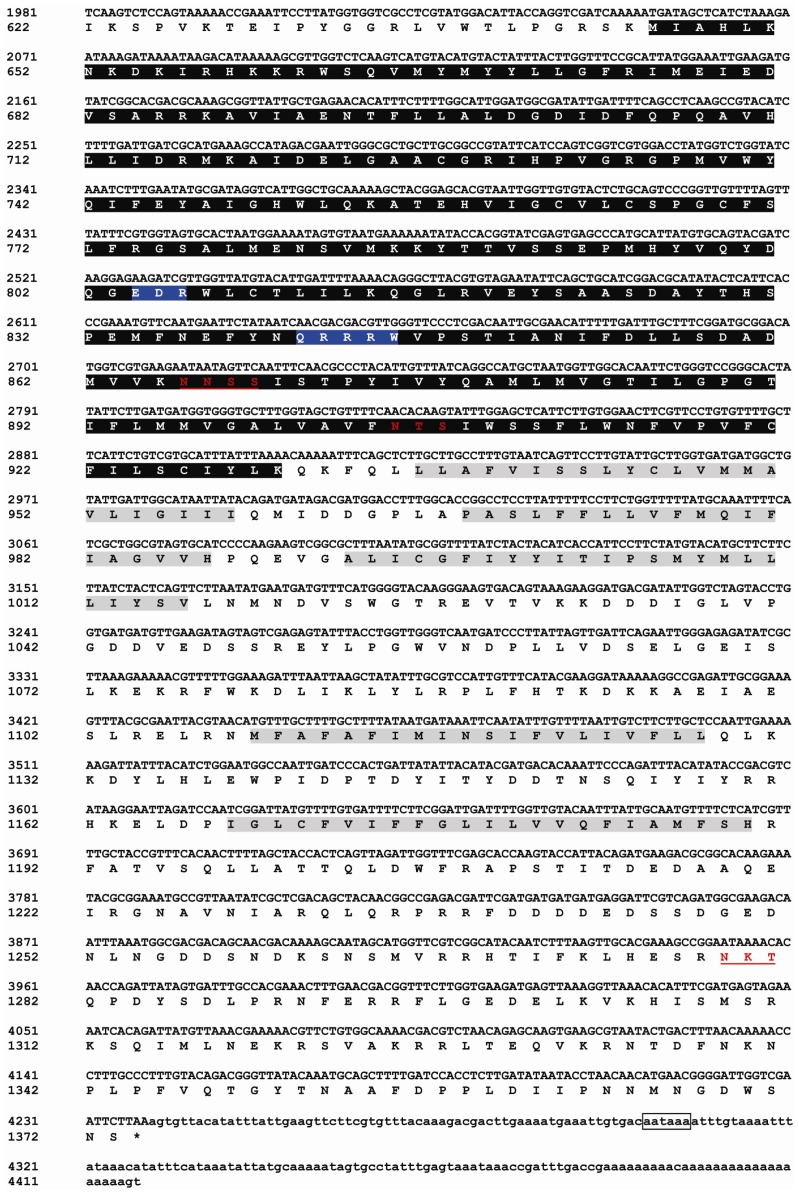
Nucleotide and deduced amino acid sequences of *BdCHS2* cDNA from *Bactrocera dorsalis* (KC354694). The start codon is indicated in bold and the stop codon in bold with an asterisk. The putative polyadenylation signal (AATAA) is boxed. The putative transmembrane regions are shaded. The five potential *N*-glycosylation sites are double underlined. The amino acid sequence of the putative catalytic domain is in gray with black background. The signature sequences (EDR and QRRRW) are in white with a wavy line.

The full-length cDNA sequence of *BdCHS2* was obtained by PCR (Polymerase Chain Reaction) and 5′ and 3′ RACE. The complete cDNA of the *BdCHS2* (GenBank ID: KC354694) consisted of 4417 nucleotides with an open reading frame (ORF) of 4122 nucleotides encoding 1373 amino acids. The cDNA included a 5′-untranslated region (UTR) located 116 nucleotides upstream of the start codon (ATG) and a 3′ UTR of 179 nucleotides ending in a poly (A) tail. The complete nucleotide and deduced amino acid sequences of *BdCHS2* were shown in Figure 1. A possible consensus signal sequence for polyadenylation (AATAAA) was located 79 nucleotides upstream of the poly (A) tail. The theoretical molecular weight of *BdCHS2* based on the deduced amino acid sequence was calculated to be 158.5 kDa, with an isoelectric point of 6.83.

*BdCHS2* was predicted to have three domains: an *N*-terminal domain (residues 1–645) with eight transmembrane helices; a catalytic domain (residues 646–930); and a *C*-terminal domain (residues 931–1373) with an additional five transmembrane helices. The signature sequence “QRRRW”, “WGTRE”, and “EDR” for chitin synthases were also found in *BdCHS2*. Five potential *N*-glycosylation sites was predicted using NetNGLyc 1.0 software (Technical University of Denmark, Copenhagen, Denmark), suggesting that the protein was glycosylated. However, no signal peptide was found.

**Figure 2:**
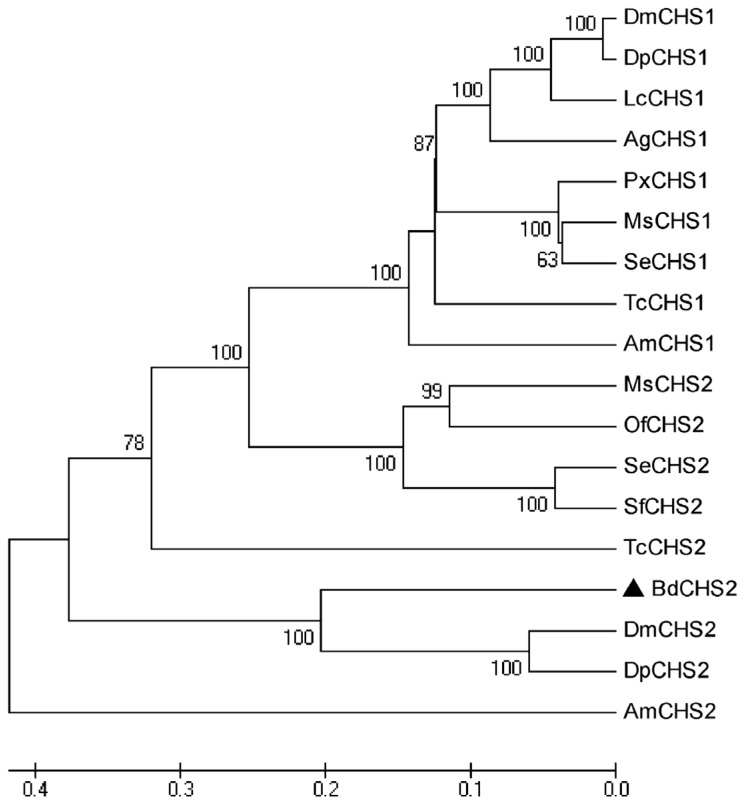
Evolutionary relationships of deduced amino acid sequence of *BdCHS2* with other insect chitin synthases constructed using the neighbor-joining method. Bootstrap values with 1000 trials are indicated on branches. The scale bar represents the number of substitutions per site. The following insect chitin synthases sequence were used: *Anopheles gambiae* (Ag), *Apis mellifera* (Am), *Bactrocera dorsalis* (Bd), *Drosophila melanogaster* (Dm), *Drosophila pseudoobscura* (Dp), *Lucilia cuprina* (Lc), *Manduca sexta* (Ms), *Ostrinia furnacalis* (Of), *Plutella xylostella* (Px), *Spodoptera exigua* (Se), *Spodoptera frugiperda* (Sf), *Tribolium castaneum* (Tc). GenBank accession numbers are as follows: *AgCHS1* (XP_321336), *AmCHS1* (XP_395677), *AmCHS2* (XP_001121152), *BdCHS2* (KC354694), *DmCHS1* (NP524233), *DmCHS2* (NP_001137997), *DpCHS1* (XP_001359390), *DpCHS2* (XP_001352881), *LcCHS1* (AF221067), *MsCHS1* (AY062175), *MsCHS2* (AY82156), *OfCHS2* (AB_B97082), *PxCHS1* (BAF47974), *SeCHS1* (DQ062153), *SeCHS2* (DQ912929), *SfCHS2* (AY525599), *TcCHS1* (AY291475), and *TcCHS2* (AY291477).

Multiple protein alignments showed that BdCHS2 protein had homology to the known and predicted CHS2 in other insect species. For instance, the BdCHS2 protein shares 87% identity with the CHS2 of *Drosophila mojavensis* (XP_002008568), 85% identity with the CHS2 of *D. persimilis* (XP_002027231), 84% identity with the CHS2 of *D. melanogaster* (NP_001137997), and 81% identity with the CHS2 of *Culex quinquefasciatus* (XP_001864594). A phylogenetic tree was constructed based on the neighbor-joining method using complete CHSs proteins deposited in NCBI by MEGA 5.04 (Figure 2). The tree showed that BdCHS2 was classified into the CHS2 family, and was most closely related to DmCHS2 and DpCHS2 with these three genes clustering together.

### 2.2. Tissue-Specific Expression Pattern of *BdCHS2*

**Figure 3:**
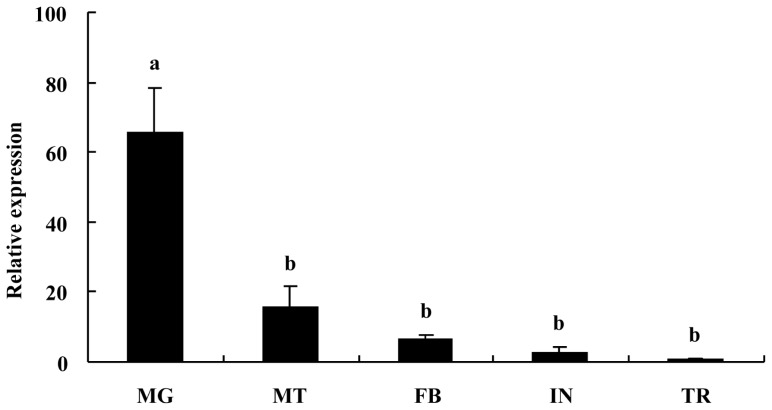
Expression profiles of the *BdCHS2* in different tissues of larval *Bactrocera dorsalis.* The tissues include integument (IN), fat body (FB), midgut (MG), Malpighian tubules (MT), and trachea (TR). α-Tubulin was used as an internal reference gene. The relative expression was calculated based on the value of the lowest expression, which was ascribed an arbitrary value of 1. Data are means ± SE of three biological replications. Different letters above each bar indicate statistically significant difference by ANOVA followed by the Duncan’s multiple range test (*p* < 0.05).

The expression of *BdCHS2* mRNA was investigated in various tissues in the third instar larvae of *B. dorsalis* (Figure 3). *BdCHS2* was highly expressed in the midgut, but detected at low levels in fat body, Malpighian tubules, integument, or trachea. The relative expression level of *BdCHS2* was the highest in midgut among the five tissues, and it was 66-, 16-, 7- and 3-fold higher in midgut, Malpighian tubule, fat body, and integument, respectively, than that in trachea.

### 2.3. *BdCHS2* Expression and Total Chitin Content during Development

**Figure 4:**
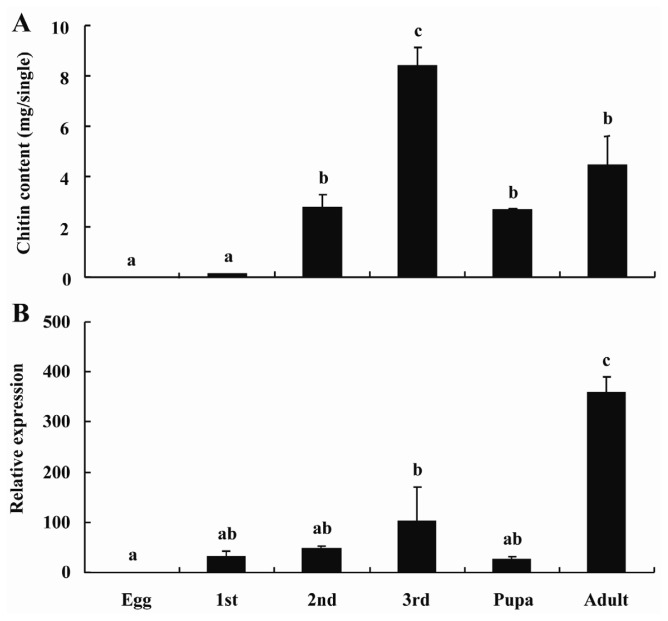
(**A**) Developmental changes of total chitin content and (**B**) mRNA levels of *BdCHS2* in *Bactrocera dorsalis*. α-Tubulin was used as an internal reference gene. The relative expression was calculated based on the value of the lowest expression, which was ascribed an arbitrary value of 1. Data are means ± SE of three biological replications. Different letters above each bar indicate statistically significant difference by ANOVA followed by the Duncan’s multiple range test (*p* < 0.05).

To understand the function of *BdCHS2*, its expression patterns during development from egg to adult were examined (Figure 4). The results showed that *BdCHS2* was expressed at all stages, indicating that it has a role throughout the entire life cycle. The highest mRNA level was found in the adult stage, and the relative expression levels of *BdCHS2* was 31-, 47-, 102-, 26- and 358-fold higher in the first, second and third instar larvae, pupa, and adult than in the egg, respectively. Subsequently, the relative expression level of *BdCHS2* in egg, the first, second and third instar larvae, and pupa were significantly lower from that in the adult (*p* < 0.05). There was an increasing expression level of *BdCHS2* during the developmental period from the egg to the third instar larvae.

The chitin content was detected from the whole bodies of *B. dorsalis* during the developmental stages. The results showed that the highest chitin content was observed in the third instar larvae and the lowest content was in the egg (Figure 4). There was a positive relationship between *BdCHS2* expression level and the total chitin contents during development.

### 2.4. Feeding-Mediated Changes in Transcript Levels of *BdCHS2* and Chitin Content in Midgut

**Figure 5:**
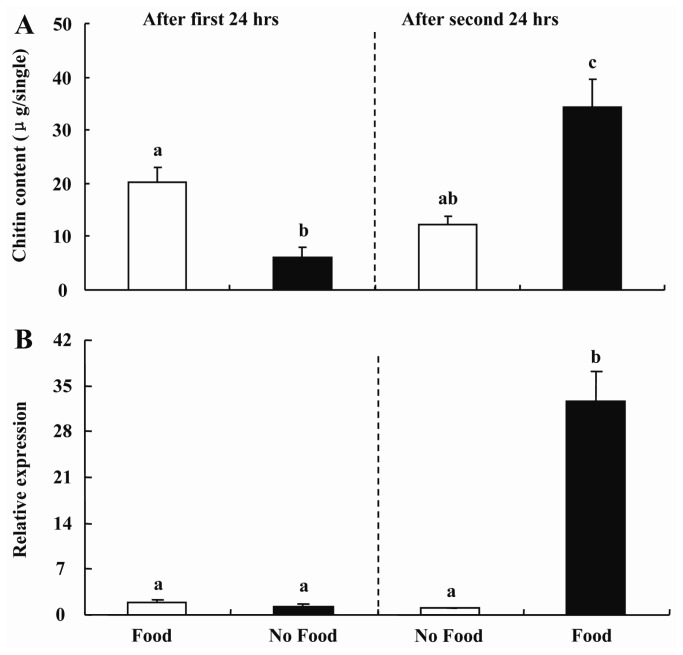
(**A**) Changes of chitin content and (**B**) mRNA levels of *BdCHS2* in the midgut of third instar larvae of *Bactrocera dorsalis* under food or no food conditions. Larvae in set 1 (empty bars) were fed for 24 h and then maintained with no food for the next 24 h, whereas larvae in set 2 (black bars) were maintained with no food for 24 h and then fed for the next 24 h. α-Tubulin was used as an internal reference gene. Data are means ± SE of four biological replications, each with two technical replications. Different letters above each bar indicate statistically significant difference by ANOVA followed by the Duncan’s multiple range test (*p* < 0.05).

Furthermore, to test the hypothesis that midgut chitin content was regulated during feeding, presumably to alter the porosity of the peritrophic membrane to facilitate food digestion, we examined the changes in transcript levels of *BdCHS2* and chitin content in the midgut of larvae *B. dorsalis* with or without food. When the larvae were maintained with food for the first 24 h, the transcript levels of *BdCHS2* in the midgut were 1.5-fold higher than that for larvae maintained with no food (*p* < 0.05). However, when the larvae maintained on food were transferred to a container with no food for another 24 h, the transcript level of *BdCHS2* decreased by 20% (*p* < 0.05). In contrast, when the larvae were maintained with no food for the first 24 h, then were transferred to a container with food for the next 24 h, the transcript level increased by 24.6-fold (*p* < 0.05) (Figure 5).

When the larvae maintained on the food were transferred to a container without food for another 24 h, the chitin content decreased by 40% (*p* < 0.05). In contrast, when the larvae maintained with no food for the first 24 h, then were transferred to a container with food for the next 24 h, the chitin content level increased by 4.5-fold (*p* < 0.05) (Figure 5). Moreover, there was a positive relationship between *BdCHS2* expression level and chitin content in the midgut.

### 2.5. Discussion

Tellam and his colleagues first isolated the complete cDNA sequence of putative chitin synthase in arthropod [21]. Two distinct *CHS* genes have been studied through molecular cloning and functional analyses in several orders in insects, such as Diptera, Orthoptera, Coleoptera, Lepidoptera, and Hymenoptera [11]. CHS was mainly responsible for the chitin synthesis in cuticular exoskeleton, tracheae and the PM in midgut. Recently, much more information about the *CHS1* gene has been studied including *B. dorsalis* [22] while relatively little information is available about the gene *CHS2* being involved in the midgut chitin synthesis in insects. In the present work, via molecular bioinformatics including sequence similarity analysis, unique signature sequences and phylogenetic analysis, it was confirmed that the sequence we cloned from the *B. dorsalis* was another chitin synthase gene *BdCHS2*. The isolation of *BdCHS2* cDNA provided us an opportunity to study the expression patterns and biological functions of this gene in *B. dorsalis*.

Furthermore, the expression profiles of *BdCHS2* in five different tissues were investigated. The results indicated that the *BdCHS2* was expressed highest in midgut which was consistent with the expression pattern of *CHS2* in other insects, including *D. melanogaster* [13], *A. gambiae* [11], *T. castaneum* [14], *M. sexta* [15], *S. exigua* [10], *O. furnacali* [16], *S. Frugiperda* [9], *L. migratoria* [17], and *A. aegypti* [19]. This result was also consistent with the hypothesis that *CHS2* was responsible for biosynthesis of the chitin in midgut. *BdCHS2* was expressed at a low level in integument and trachea which might be associated with *CHS1* of its chitin biosynthesis [22,23–26]. However, in *A. gambiae*, CHS2 protein was detected not only in the midgut, but also in newly formed compound eyes and abdominal inter-segmental regions of the pupae [11]. In *A. aegypti*, CHS2 localized to the periphery of the epithelial cells facing the midgut lumen [12]. Equally, the anterior midgut may play an important role in chitin biosynthesis more than the rest of the midgut in *L. migratoria* [17]. In summary, the *CHS2* gene is mainly expressed in midgut and much more function of this gene is necessary for further research.

The chitin content and the *BdCHS2* expression level were investigated in this study, and a similar trend was found during development except for the adult stage. This result was consistent with a recent study, *i.e.*, the expression of *LmCHS2* gradually increased from first to fifth-instar nymphs, and reached the highest in the first day of adults in *L. migratoria* [17]. In *S. exigua*, the expression level in different developmental stages also showed a similar trend to that found in our present study [10]. In *S. frugiperda*, *SfCHS2* expressed in the midgut during the feeding stages [9] was also consistent with our results. High expression levels of *BdCHS2* during the feeding stage indicated that BdCHS2 protein plays an important role in the production of the chitin-rich PM. The insect needs this structure to protect the gut lining cells and increase the efficiency of nutrient digestion during feeding stages [27,28]. Additionally, the trend of gene expression level had a positive correlation with that of total chitin content during development stages, indicating that this gene may play an important role in total body chitin synthesis.

Furthermore, we examined the changes in transcript levels of *BdCHS2* and midgut chitin content in larvae of *B. dorsalis* fed on the artificial diet or starvation. Our results suggested that the expression level of the *BdCHS2* was affected by feeding and this was in agreement with the report in blood-fed insects *A. gambiae* [29] and *L. longipalpis* [30]. In contrast, in *Ostrinia nubilalis*, expression level changes of *CHS2* had a completely opposite result and chitinase had a similar result [18]. It might be due to the significant differences in the biological habits of these two insects, which belong to different Orders. Furthermore, their type of PM belong to two different types, *O. nubilalis* belongs to type I while *B. dorsalis* to type II PM [6]. In the present study, the chitin content of the midgut dissected from the larvae showed positive proof of a consistent correlation with change in gene expression level. From expression profiles of tissue and developmental stages, we can infer that *BdCHS2* was mainly expressed in midgut and had a gradually increased expression level from the second instar to the third instar larvae. However, the expression level of *BdCHS2* and the chitin content of the midgut decreased after treatment with food for 24 h then starvation for 24 h, indicating that starvation had a strong influence on expression of this gene in the midgut. On the other hand, Chironomid larvae only break down newly assimilated food for energy during starvation [31]. Therefore, the reason why the chitin content decreased after 24 h starvation may be that *BdCHS2* was expressed at a low level of mRNA in midgut; additionally, the midgut chitin might be degraded to survive during the period of starvation. As expected, under the condition of feeding for 24 h after starvation for the first 24 h, gene expression and the chitin content level increased rapidly. It may be that the body needs much more digested food to grow into later developmental stages along with the increased midgut chitin and the mRNAs of *BdCHS2* after the starvation for 24 h. The hypothesis that the midgut chitin content level is regulated during feeding, presumably to facilitate food digestion, was confirmed. In brief, the change under the feeding and starvation conditions suggested that *BdCHS2* plays important roles in the regulation of chitin contents in the midgut. By using RNAi methodologies, it has been shown that the insect ceased feeding, shrinked in larval size, decreased in midgut chitin content [20], exhibited a high mortality [17], and disrupted formation of the peritrophic matrix [19] after *CHS2* gene knockdown. Moreover, transgenic plants synthesized hairpin dsRNAs as a protective measure against damaging herbivorous insects [32]. Based on the results of RNAi in other insects and the results in this study, *BdCHS2* might be a good candidate gene for *B. dorsalis* control by transgenic plants due to the ability to suppress a gene critical for insect survival, providing a new approach to block a significant pest using environmentally friendly and effective principles.

## 3. Experimental Section

### 3.1. Test Insect

The colony of *B. dorsalis* was kept in laboratory cages at 27 ± 1 °C, 70% ± 5% relative humidity and a photoperiod cycle of 14 h·Light/10 h·Dark. The insects were reared on an artificial diet as described previously [33]. The developmental stages were synchronized at each egg incubation. Fat body, integument, Malpighian tubules, midgut, and trachea were dissected from the third instar larvae in phosphate buffered saline (PBS) under a stereomicroscope (Olympus SZX12, Tokyo, Japan) and stored at −80 °C prior to use.

### 3.2. cDNA Cloning of *BdCHS2* and Sequence Analysis

#### 3.2.1. RNA Extraction and cDNA Synthesis

Total RNA was extracted from the midgut of the third instar larvae of *B. dorsalis* with TRIzol Reagent (Invitrogen, Carlsbad, CA, USA) according to the manufacturer’s instructions, and used in the amplification of cDNA fragments and rapid amplification of cDNA ends (RACE). The total RNA was treated with DNase (TaKaRa, Dalian, China) and dissolved in 30 μL DEPC treated water. The purity and quantity of extracted RNA was quantified by the ratio of OD_260_/OD_280_ with an ultraviolet spectrometer. First-strand cDNA was synthesized from 2 μg of DNase-treated RNA by PrimeScript^®^ 1st Strand cDNA synthesis Kit (TaKaRa, Ohtsu, Japan) with oligo (dT)_18_ primers, and used as a template for PCR.

#### 3.2.2. Obtaining Full-Length of *BdCHS2* cDNA

**Figure 6:**
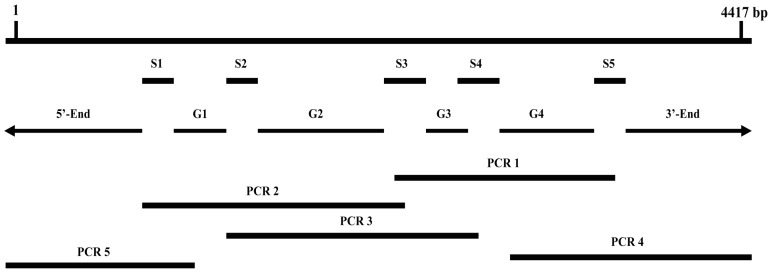
PCR amplification and cloning of the full-length *BdCHS2* cDNA in *Bactrocera dorsalis*. Five PCR fragments (S1–S5) were generated from a transcriptome sequencing data of *B. dorsalis*. Based on S1–S5 sequences, four gaps (G1–G4) were amplified. The 3′- and 5′-end fragments were obtained through 3′- and 5′-RACE respectively. PCR1–PCR5 fragments were amplified with specific primers designed according to the assembled full-length cDNA sequences of *BdCHS2*.

Based on the transcriptome sequencing data of *B. dorsalis* [34], five cDNA fragments encoding *BdCHS2* (S1–S5) were identified (Table 1). In order to generate a larger cDNA fragment, three pairs of primers (Table 2) were designed to amplify the three gaps among the assembled fragments of *BdCHS2* (PCR1 to PCR3, Figure 6). 3′- and 5′-RACE ends (PCR4 and PCR5) were amplified according to the instructions of SMARTer™ RACE cDNA Amplification Kit (Clontech, Palo Alto, CA, USA). PCR amplifications were carried out in a total volume of 25 μL mixture, containing 2.5 μL Mg^2+^ (2.5 mM), 2 μL dNTPs (2.5 mM), 2.5 μL 10× PCR Buffer (Mg^2+^ free), 1 μL each primer (10 mM), 1 μL cDNA, and 0.25 μL rTaq™ polymerase (TaKaRa), and 15 μL ddH_2_O. Thermal cycling conditions were 95 °C for 5 min followed by 34 cycles of 95 °C for 30 s, 58 °C for 30 s and 72 °C for 1 min. The last cycle was followed by final extension at 72 °C for 10 min. The amplified products were analyzed on 1.0% agarose gel, which contained GoodView™ (SBS Genetech, Beijing, China). The target band of products was purified using the Gel Extraction Mini Kit (Watson Biotechnologies, Shanghai, China). Purified DNA was ligated into pGEM^®^-T Easy vector (Promega, Madison, WI, USA). The ligation reactions were transformed into Trans-T1 competent cells (Transgen, Beijing, China). By using standard ampicillin selection, successful clones were picked out and then PCR with gene-specific primers, and further sequenced in both directions with an ABI Model 3100 automated sequencer (BGI, Shenzhen, China).

#### 3.2.3. Sequence Analysis and Phylogenetic Tree Construction

Searching for similar sequences was performed using BlastP in the non-redundant protein sequences (nr) database of the NCBI website [35]. The open reading frame (ORF) finder tool at the NCBI was used to identify the ORF of *BdCHS2*. Sequences were edited with DNAMAN 5.2.2 (Lynnon BioSoft, Quebec, Canada). ExPASy Proteomics Server [36] was used to compute isoelectric point and molecular weight of the deduced protein sequences. NetNGlyc 1.0 Server [37] was used to analyze the *N*-glycosylation sites. Cellular localization was conducted with the web site [38]. The signal peptide was predicted by SignalP 3.0 [39], and transmembrane helices were analyzed using TMHMM v.2.0 [40]. The neighbor-joining method was applied to construct a phylogenetic tree with 1000 replications as the bootstrap value using MEGA 5.04 [41].

### 3.3. Tissue-Specific Expression of *BdCHS2* Using Quantitative Real-Time PCR

Tissue-specific expression of *BdCHS2* was examined by quantitative real-time PCR (qPCR). Total RNA was isolated from fat body, integument, Malpighian tubules, midgut, and trachea of the third instar larvae, using RNeasy^®^ Plus Micro Kit (with gDNA Elimator spin columns, Qiagen, Valencia, CA, USA). First strand cDNA was synthesized in a 10 μL reaction mixture using random hexamers by PrimeScript^®^ RT reagent Kit (TaKaRa). The qPCR was conducted on Mx3000P thermal cycler (Stratagene, La Jolla, CA, USA) using SYBR Green detection system (iQ™ SYBR^®^ Green Supermix, BIO-RAD, Hercules, CA, USA) and gene-specific primers CHS2-Q-F and CHS2-Q-R (Table 2). The PCR amplifications were performed in 20 μL reaction systems, including 7 μL ddH_2_O, 10 μL SYBR Green Supermix, 1 μL of template cDNA and 1 μL of each primer (0.2 mM) under the following conditions: pre-denaturation at 95 °C for 2 min, 40 cycles of denaturation at 95 °C for 15 s, annealing at 60 °C for 30 s, and elongation at 72 °C for 30 s. After reaction, a melting curve analysis from 60 to 95 °C was applied to all reactions to ensure consistency and specificity of the amplified. The qPCR analysis had three times of biological duplication. The data were normalized to the stable reference gene α-Tubulin (GU269902) (Table 2) based on our previous evaluations, and was calculated using 2^−ΔΔCT^ method [42].

### 3.4. Developmental Stages-Specific Expression of *BdCHS2* and Total Chitin Content

Eggs, the first, second, and third instar larvae, pupae, and adults were used for total RNA isolation using RNeasy^®^ Plus Micro Kit (with gDNA Elimator spin columns, Qiagen, Valencia, CA, USA) (e.g., egg, the first instar larvae) or TRIzol reagent and treated with DNase (TaKaRa) for DNA digestion (e.g., the second, and third instar larvae, pupae, and adults). The stage-specific expression was examined using qPCR as pre-mentioned method. Furthermore, the chitin content in different developmental stages was assayed based on the previous described method [43–45]. Briefly, the sample (30 individuals for each sample) was homogenized with 1.0 mL of distilled water by grinding in a cold mortar. Then, the chitin was isolated from the sample after treated by centrifuged and 3% SDS (sodium dodecyl sulfate). To deacetylate chitin, it was re-suspended in 0.3 mL of 14 M KOH and incubated in drying oven at 130 °C for 1 h. The insoluble chitosan was obtained after purified by different concentrations of alcohol. 100 μL of the chitosan solution was mixed with 100 μL of 10% NaNO_2_ and 100 μL of 10% KHSO_4_ to depolymerize the chitosan and deaminate the glucosamine residues from the chitosan. After treated by 12.5% NH_4_SO_3_NH_2_ (Sigma-Aldrich, St. Louis, MO, USA), the sample was added to MBTH (3-methyl-2-benzothiazolone hydrazone hydrochloride hydrate, Sigma-Aldrich) (50 mg/10 mL) and 0.83% FeCl_3_. Finally, 100 μL of each sample was transferred to a 96-well microplate and then colorimetric assay under 650 nm in a microplate reader (Sigma Laborzentrifugen GmbH, Ostrode, Germany). According to a standard curve constructed by using known concentrations of glucosamine (Sigma-Aldrich), chitin content was calculated as a glucosamine equivalent. Three biological replications, each with two technical replications, were used in this analysis.

### 3.5. Gene Expression Profiles and Chitin Content Assay under Feeding and Starvation Conditions

The 1-day-old third instar larvae were used for this experiment. Eight Petri dishes (diameter = 4 cm) were divided into two groups, each with four Petri dishes. The insects in the first group were maintained with the artificial diet (designated as with food) for 24 h and then with no food for next 24 h, while the insects in the second group were maintained with no food for 24 h and then with food for the next 24 h. Total RNA was isolated from the dissected midguts of the two groups after 24 and 48 h treatment. The transcript levels were measured using qPCR as mentioned above. Furthermore, the chitin content in the midguts of the above treated larvae was assayed.

## 4. Conclusions

In conclusion, a full-length cDNA encoding chitin synthase 2 was obtained from *B. dorsalis. BdCHS2* was mainly expressed in midgut. Further, it expressed in all developmental stages, while highly in the feeding stages (larval and adult stage), and also had a positive relation to the total chitin content of the insect. In addition, the feeding and starvation had a very important effect on this gene expression. In sum, *BdCHS2* is involved in the regulation of the midgut chitin and subsequently affects the growth and development of *B. dorsalis*.

## Figures and Tables

**Table 1:** The cDNA fragments of *BdCHS2* extracted from a transcriptome sequencing data of *B. dorsalis*.

cDNA fragment	Length (bp)	Position in the coding area of *BdCHS2* (bp)
S1	284	770–1,053
S2	183	1,353–1,535
S3	319	2,177–2,495
S4	252	2,643–2,894
S5	243	3,718–3,960

**Table 2:** Primers used in this study.

Application of primers	cDNA fragment	Primer name	Primer sequence(5′-3′)	cDNA position in the coding area (bp)
cDNA cloning	PCR 1	CHS2-1	TACTCTGCAGTCCCGGTTGTT	2404–2424
		CHS2-2	CTTGTGCCGCGTCTTCATCTG	3757–3777

	PCR 2	CHS2-3	TAGTCGTTCTAGATATCAGAC	926–946
		CHS2-4	AGCAGCGCCCAATTCGTCTATG	2273–2294

	PCR 3	CHS2-5	GGATAACTCGACATATTTGGC	1465–1485
		CHS2-6	TGTAGGGCGTTGAAATTGAACTA	2717–2739

	PCR 4 (3′-RACE)	CHS2-7	GGAAGTGACAGTAAAGAAGGATG	3197–3219
		CHS2-8	TAAATGGCGACGACAGCAACG	3874–3894

	PCR 5 (5′-RACE)	CHS2-9	CCACATAGCAACGGCAACAGAAGC	1290–1313
		CHS2-10	TAATGGGAGGGCGATTATTTGTAAC	821–845

		UPM	CTAATACGACTCACTATAGGGC	–
		NUP	AAGCAGTGGTATCAACGCAGAGT	–

qPCR analysis	CHS2	CHS2-Q-F	ATTTTCAGCCTCAAGCCGTA	2227–2246
		CHS2-Q-R	CGGGACTGCAGAGTACACAA	2399–2418

	A-tubulin	α-tub-F	CGCATTCATGGTTGATAACG	–
		α-tub-R	GGGCACCAAGTTAGTCTGGA	–

## References

[1] Stephens A., Kriticos D., Leriche A. (2007). The current and future potential geographical distribution of the oriental fruit fly, *Bactrocera dorsalis* (Diptera: Tephritidae). Bull. Entomol. Res.

[2] Clarke A.R., Armstrong K.F., Carmichael A.E., Milne J.R., Raghu S., Roderick G.K., Yeates D.K. (2005). Invasive phytophagous pests arising through a recent tropical evolutionary radiation: The *Bactrocera dorsalis* complex of fruit flies. Annu. Rev. Entomol.

[3] Hsu J.C., Feng H.T., Wu W.J., Geib S., Mao C.H., Vontas J. (2012). Truncated transcripts of nicotinic acetylcholine subunit gene Bdα6 are associated with spinosad resistance in *Bactrocera dorsalis*. Insect Biochem. Mol. Biol.

[4] Jin T., Zeng L., Lin Y., Lu Y., Liang G. (2011). Insecticide resistance of the oriental fruit fly, *Bactrocera dorsalis* (Hendel) (Diptera: Tephritidae), in mainland China. Pest Manag. Sci.

[5] Kramer K., Muthukrishnan S., Gilbert L.I., Iatrou K., Gill S. (2005). Chitin Metabolism in Insects. Comprehensive Molecular Insect Science.

[6] Lehane M. (1997). Peritrophic matrix structure and function. Annu. Rev. Entomol.

[7] Merzendorfer H. (2006). Insect chitin synthases: A review. J. Comp. Physiol. B Biochem. Syst. Environ. Physiol.

[8] Arakane Y., Specht C.A., Kramer K.J., Muthukrishnan S., Beeman R.W. (2008). Chitin synthases are required for survival, fecundity and egg hatch in the red flour beetle, *Tribolium castaneum*. Insect Biochem. Mol. Biol.

[9] Bolognesi R., Arakane Y., Muthukrishnan S., Kramer K.J., Terra W.R., Ferreira C. (2005). Sequences of cDNAs and expression of genes encoding chitin synthase and chitinase in the midgut of *Spodoptera frugiperda*. Insect Biochem. Mol. Biol.

[10] Kumar N.S., Tang B., Chen X., Tian H., Zhang W. (2008). Molecular cloning, expression pattern and comparative analysis of chitin synthase gene B in *Spodoptera exigua*. J. Comp. Physiol. B.

[11] Zhang X., Zhang J., Park Y., Zhu K.Y. (2012). Identification and characterization of two chitin synthase genes in African malaria mosquito, *Anopheles gambiae*. Insect Biochem. Mol. Biol.

[12] Ibrahim G.H., Smartt C.T., Kiley L.M., Christensen B.M. (2000). Cloning and characterization of a chitin synthase cDNA from the mosquito *Aedes aegypti*. Insect Biochem. Mol. Biol.

[13] Gagou M.E., Kapsetaki M., Turberg A., Kafetzopoulos D. (2002). Stage-specific expression of the chitin synthase *DmeChSA* and *DmeChSB* genes during the onset of *Drosophila* metamorphosis. Insect Biochem. Mol. Biol.

[14] Arakane Y., Hogenkamp D.G., Zhu Y.C., Kramer K.J., Specht C.A., Beeman R.W., Kanost M.R., Muthukrishnan S. (2004). Characterization of two chitin synthase genes of the red flour beetle, *Tribolium castaneum*, and alternate exon usage in one of the genes during development. Insect Biochem. Mol. Biol.

[15] Hogenkamp D.G., Arakane Y., Zimoch L., Merzendorfer H., Kramer K.J., Beeman R.W., Kanost M.R., Specht C.A., Muthukrishnan S. (2005). Chitin synthase genes in *Manduca sexta*: Characterization of a gut-specific transcript and differential tissue expression of alternately spliced mRNAs during development. Insect Biochem. Mol. Biol.

[16] Qu M., Liu T., Yang J., Yang Q. (2011). The gene, expression pattern and subcellular localization of chitin synthase B from the insect *Ostrinia furnacalis*. Biochem. Biophys. Res. Commun.

[17] Liu X., Zhang H., Li S., Zhu K.Y., Ma E., Zhang J. (2012). Characterization of a midgut-specific chitin synthase gene (*LmCHS2*) responsible for biosynthesis of chitin of peritrophic matrix in *Locusta migratoria*. Insect Biochem. Mol. Biol.

[18] Khajuria C., Buschman L.L., Chen M.S., Muthukrishnan S., Zhu K.Y. (2010). A gut-specific chitinase gene essential for regulation of chitin content of peritrophic matrix and growth of *Ostrinia nubilalis* larvae. Insect Biochem. Mol. Biol.

[19] Kato N., Mueller C.R., Fuchs J.F., Wessely V., Lan Q., Christensen B.M. (2006). Regulatory mechanisms of chitin biosynthesis and roles of chitin in peritrophic matrix formation in the midgut of adult *Aedes aegypti*. Insect Biochem. Mol. Biol.

[20] Arakane Y., Muthukrishnan S., Kramer K.J., Specht C.A., Tomoyasu Y., Lorenzen M.D., Kanost M., Beeman R.W. (2005). The *Tribolium* chitin synthase genes *TcCHS1* and *TcCHS2* are specialized for synthesis of epidermal cuticle and midgut peritrophic matrix. Insect Biochem. Mol. Biol.

[21] Tellam R.L., Vuocolo T., Johnson S.E., Jarmey J., Pearson R.D. (2000). Insect chitin synthase-cDNA sequence, gene organization and expression. Eur. J. Biochem.

[22] Yang W.J., Xu K.K., Cong L., Wang J.J. (2013). Identification, mRNA expression, and functional analysis of chitin synthase 1 gene and its two alternative splicing variants in oriental fruit fly, *Bactrocera dorsalis*. Int. J. Biol. Sci.

[23] Wang Y., Fan H.W., Huang H.J., Xue J., Wu W.J., Bao Y.Y., Xu H.J., Zhu Z.R., Cheng J.A., Zhang C.X. (2012). Chitin synthase 1 gene and its two alternative splicing variants from two sap-sucking insects, *Nilaparvatalugens* and *Laodelphaxstriatellus* (Hemiptera: Delphacidae). Insect Biochem. Mol. Biol.

[24] Qu M., Yang Q. (2011). A novel alternative splicing site of class A chitin synthase from the insect *Ostrinia furnacalis*-Gene organization, expression pattern and physiological significance. Insect Biochem. Mol. Biol.

[25] Ampasala D.R., Zheng S., Zhang D., Ladd T., Doucet D., Krell P.J., Retnakaran A., Feng Q. (2011). An epidermis-specific chitin synthase cDNA in *Choristoneura fumiferana*: Cloning, characterization, developmental and hormonal-regulated expression. Arch. Insect Biochem. Physiol.

[26] Zhang J., Liu X., Li D., Sun Y., Guo Y., Ma E., Zhu K.Y. (2010). Silencing of two alternative splicing-derived mRNA variants of chitin synthase 1 gene by RNAi is lethal to the oriental migratory locust, *Locusta migratoria manilensis* (Meyen). Insect Biochem. Mol. Biol.

[27] Terra W.R. (2001). The origin and functions of the insect peritrophic membrane and peritrophic gel. Arch. Insect Biochem. Physiol.

[28] Terra W., Ferreira C. (2005). Biochemistry of digestion. Compr. Mol. Insect Sci.

[29] Shen Z., Jacobs-Lorena M. (1997). Characterization of a novel gut-specific chitinase gene from the human malaria vector *Anopheles gambiae*. J. Biol. Chem.

[30] Ramalho-Ortigao J., Traub-Csekö Y. (2003). Molecular characterization of *Llchit1*, a midgut chitinase cDNA from the leishmaniasis vector *Lutzomyia longipalpis*. Insect Biochem. Mol. Biol.

[31] Kikuchi E., Takagi S., Shikano S. (2007). Changes in carbon and nitrogen stable isotopes of chironomid larvae during growth, starvation and metamorphosis. Rapid Commun. Mass Spectrom.

[32] Baum J.A., Bogaert T., Clinton W., Heck G.R., Feldmann P., Ilagan O., Johnson S., Plaetinck G., Munyikwa T., Pleau M. (2007). Control of coleopteran insect pests through RNA interference. Nat. Biotechnol.

[33] Cong L., Yang W.J., Shen G.M., Dou W., Wang J.J. (2012). Molecular characterization of the cDNA encoding ecdysone receptor isoform B1 and its expression in the oriental fruit fly, *Bactrocera dorsalis* (Diptera: Tephritidae). Fla. Entomol.

[34] Shen G.M., Dou W., Niu J.Z., Jiang H.B., Yang W.J., Jia F.X., Hu F., Cong L., Wang J.J. (2011). Transcriptome analysis of the oriental fruit fly (*Bactrocera dorsalis*). PLoS One.

[35] National Center for Biotechnology Information http://www.ncbi.nlm.nih.gov/.

[36] Compute pI/Mw Tool http://cn.expasy.org/tools/pi_tool.html.

[37] NetNGlyc 1.0 Server http://www.cbs.dtu.dk/services/NetNGlyc/.

[38] PSORT WWW Server http://psort.nibb.ac.jp/.

[39] SignalP 3.0 Server http://www.cbs.dtu.dk/services/SignalP-3.0/.

[40] TMHMM Server 2.0 http://www.cbs.dtu.dk/services/TMHMM-2.0/.

[41] Tamura K., Peterson D., Peterson N., Stecher G., Nei M., Kumar S. (2011). MEGA5: Molecular evolutionary genetics analysis using maximum likelihood, evolutionary distance, and maximum parsimony methods. Mol. Biol. Evol.

[42] Schmittgen T.D., Livak K.J. (2008). Analyzing real-time PCR data by the comparative CT method. Nat. Protoc.

[43] Lehmann P.F., White L.O. (1975). Chitin assay used to demonstrate renal localization and cortisone-enhanced growth of *Aspergillus fumigatus* Mycelium in mice. Am. Soc. Microbiol.

[44] Zhang J., Zhu K.Y. (2006). Characterization of a chitin synthase cDNA and its increased mRNA level associated with decreased chitin synthesis in *Anopheles quadrimaculatus* exposed to diflubenzuron. Insect Biochem. Mol. Biol.

[45] Ride J., Drysdale R. (1972). A rapid method for the chemical estimation of filamentous fungi in plant tissue. Physiol. Plant Pathol.

